# Cryogenic Exfoliation of 2D Stanene Nanosheets for Cancer Theranostics

**DOI:** 10.1007/s40820-021-00619-1

**Published:** 2021-03-10

**Authors:** Jiang Ouyang, Ling Zhang, Leijiao Li, Wei Chen, Zhongmin Tang, Xiaoyuan Ji, Chan Feng, Na Tao, Na Kong, Tianfeng Chen, You-Nian Liu, Wei Tao

**Affiliations:** 1 The First Affiliated Hospital, Department of Chemistry, Jinan University, Guangzhou, 510632 Guangdong People’s Republic of China; 2 Center for Nanomedicine and Department of Anesthesiology, Brigham and Women’s Hospital, Harvard Medical School, Boston, MA 02115 USA; 3 College of Chemistry and Chemical Engineering, Central South University, Changsha, 410083 Hunan People’s Republic of China; 4 School of Chemistry and Environmental Engineering, Changchun University of Science and Technology, Changchun, 130022 Jilin Province People’s Republic of China

**Keywords:** Stanene, Two-dimensional, Cryogenic exfoliation, Cancer theranostics, Nanomedicine

## Abstract

Stanene (Sn)-based materials have been extensively applied in industrial production and daily life, but their potential biomedical application remains largely unexplored, which is due to the absence of the appropriate and effective methods for fabricating Sn-based biomaterials. Herein, we explored a new approach combining cryogenic exfoliation and liquid-phase exfoliation to successfully manufacture two-dimensional (2D) Sn nanosheets (SnNSs). The obtained SnNSs exhibited a typical sheet-like structure with an average size of ~ 100 nm and a thickness of ~ 5.1 nm. After PEGylation, the resulting PEGylated SnNSs (SnNSs@PEG) exhibited good stability, superior biocompatibility, and excellent photothermal performance, which could serve as robust photothermal agents for multi-modal imaging (fluorescence/photoacoustic/photothermal imaging)-guided photothermal elimination of cancer. Furthermore, we also used first-principles density functional theory calculations to investigate the photothermal mechanism of SnNSs, revealing that the free electrons in upper and lower layers of SnNSs contribute to the conversion of the photo to thermal. This work not only introduces a new approach to fabricate 2D SnNSs but also establishes the SnNSs-based nanomedicines for photonic cancer theranostics. This new type of SnNSs with great potential in the field of nanomedicines may spur a wave of developing Sn-based biological materials to benefit biomedical applications.

**Supplementary Information:**

The online version contains supplementary material available at 10.1007/s40820-021-00619-1.

## Highlights

- 2D Sn nanosheets (SnNSs) were prepared through the combination of cryogenic exfoliation and liquid-phase exfoliation.
- The functionalized 2D SnNSs have good stability, superior biocompatibility, high photothermal conversion efficiency, and multimode imaging capability.

**Supplementary Information:**

The online version contains supplementary material available at 10.1007/s40820-021-00619-1.

## Graphical abstract

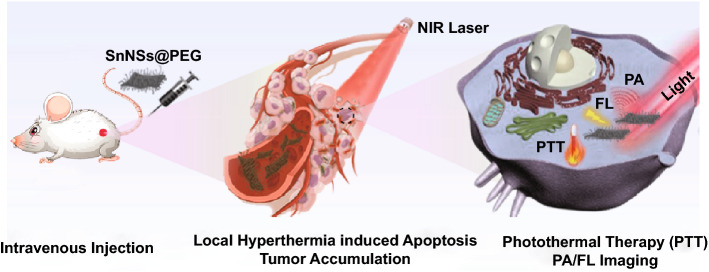

## Introduction

Stannum (Sn), one of the most commonly used metal elements ever thousands of years, is an environmentally friendly, non-toxic, antimicrobial, purified, fresh-kept, and indispensable trace element in life system and has been widely applied in people’s daily life and industrial applications [1–5]. Stannum has not only been shown great practical application value, such as developing as tableware, food packaging foil, or corrosion protection membrane for food preservation [6, 7], but also plays an essential role in the human body, which has an important influence on people’s physiological activities and maintenance of human health [1, 8]. Researches have shown that stannum can be converted into stannum compounds with antitumor activity in the human thymus, inhibiting the growth of cancer cells [9–11]. Additionally, stannum can also promote the synthesis of proteins and nucleic acids, which is beneficial to the body’s growth and development [12, 13]. The formation of some enzymes and the biological reactions of flavin enzymes also need the participation of stannum, thereby enhancing the stability of the internal environment [13, 14]. In general, stannum is a compound with a high valence state to exert its effect, in which + 2 and + 4 are the dominant valences [15–17]. The flexible variable valence allows stannum to be developed into different types of stannum-based materials, which have been widely used in various fields such as biomedicine, sensors, optoelectronics, and catalysis [18–21]. However, the diversity of stannum-based materials does not increase the physical and chemical properties of stannum materials, which hinders the interdisciplinary research of stannum-based materials.

As a typical topological 2D material, 2D Sn significantly differs from stannum-based materials such as stannum protoxide and stannum oxide, characterizing particular physicochemical properties such as tunable bandgaps, chiral phases, strong spin–orbit coupling, and quantum spin Hall effect via its stable buckled structures [22]. Until now, the main synthesis method of Sn material is to deposit Sn onto various substrates through physical vapor deposition (PVD) [22]. Zhu et al. [23] reported the first successful preparation of 2D SnNSs on the surface of Bi_2_Te_3_(111) substrate via molecular beam epitaxy (MBE), paving the avenue for further experimental studies. Moreover, some other researches also have achieved Sn growth on semiconductor Si(111) and InSb(111) substrate [24, 25], metal substrates (e.g., Pt, Au, Ag, Cu, Al, Ni, Pd, Ir) [22], and semimetallic Sb(111) substrate [26] under ultrahigh vacuum (UHV) ambiance. Nevertheless, these prepared 2D Sn materials through epitaxial growth approaches suffer from some drawbacks, such as vertical scale inhomogeneity and scalable synthesis limitation [27]. Meanwhile, this bottom-up approach to synthesize 2D Sn faces one unsolvable problem is that it is greatly difficult to transfer them from the growth substrates and use them for practical applications [27]. Although some other methods such as using ultra-fast femtosecond laser or dealloying strategy also successfully fabricate 2D SnNSs [28, 29], the cumbersome operations, expensive costs, and the large size of obtained Sn seriously limit the further applications, especially the biological applications of 2D Sn. At present, experimental efforts to synthesize 2D Sn through chemical manipulation and use it in practical applications are still in the stage of optimization morphology, mass production, and miniaturization. Although some progress has been made in the study of the thermoelectricity properties of Sn in recent years [30, 31], the application of 2D Sn nanomaterials in biomedicine is still at the beginning point. Given the unique merits including the high biosafety and clinical potential of stannum mentioned above, it is highly desired to explore a new approach to synthesize 2D Sn with nanosizes for biomedical applications.

Notably, Sn itself has an interesting phenomenon called “tin pest,” which has been known for thousands of years [32–34]. The main characteristic of this phenomenon is that pure Sn will undergo a crystalline phase transition at a temperature below 13.2 °C [33]. But the kinetic of “tin pest” at this temperature is very dilatory, and the maximum rate of “tin pest” formation commonly occurs at − 30 to − 40 °C according to the previous report [33]. During the transition, Sn is structurally fragile and readily collapses into powder, while the crystal structure of Sn in this phase is highly similar to that of silicene and germanene. This unique feature makes exfoliation of bulk Sn into 2D SnNSs through external force at ultralow temperature infinitely possibilities [27]. Additionally, liquid-phase exfoliation is also a classic top-down method of preparing 2D nanomaterials, and many 2D nanomaterials fabricated through liquid-phase exfoliation have been reported [35–39]. Our group also synthesized some 2D nanomaterials such as black phosphorus [40–44], antimonene [45, 46], and germanene [47, 48] by the liquid-phase exfoliation method. Given the general applicability of liquid-phase exfoliation and unique “tin pest” phenomenon, as well as highly similar structural characteristics between Sn and other homologous nanomaterials such as germanene and silicene [49, 50], we thus speculate that combination of cryogenic exfoliation and liquid-phase exfoliation also can be applied to the preparation of 2D SnNSs.

**Scheme 1:**
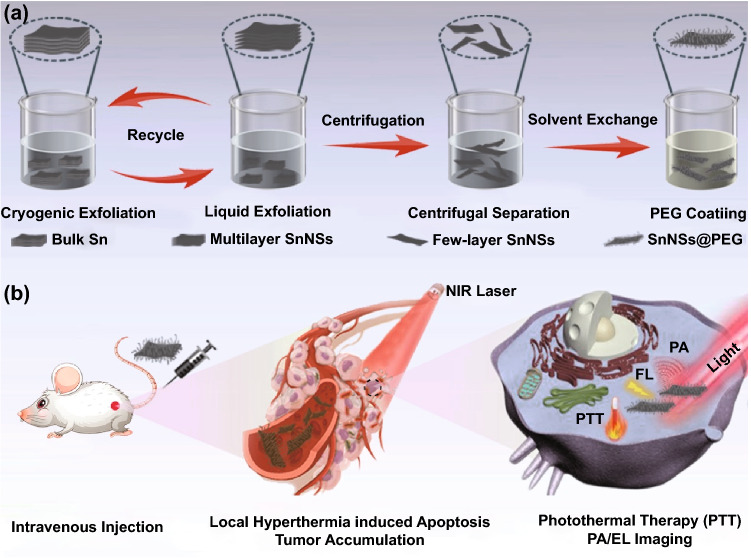
**a** Schematic diagram of the synthetic procedure of 2D SnNSs. **b** Multifunction of SnNSs@PEG in vivo

Herein, a facile top-down approach combining cryogenic exfoliation and liquid-phase exfoliation was proposed to fabricate 2D SnNSs in ethanol solution (Scheme 1a). The preparation of 2D SnNSs only requires 1.5 h in alternate atmospheres of liquid nitrogen and ambient temperature, which is far less than the time requires for typical 2D nanomaterials (e.g., black phosphorus, germanene, antimonene), greatly reducing the time cost of preparing 2D nanomaterials. After PEGylation of 2D SnNSs, the obtained SnNSs@PEG exhibited prominent photothermal performance, superior biocompatibility, and favorable stability. Simultaneously, the engineered SnNSs@PEG also have the features of multi-mode imaging (NIR fluorescence/photoacoustic/photothermal imaging), which can guide the in vivo cancer therapy in real time and noninvasively (Scheme 1b). To the best of our knowledge, this is the first proof of principle for the fabrication of 2D SnNSs using cryogenic exfoliation and liquid-phase exfoliation, and the obtained 2D SnNSs were further developed into photonic nanomedicine for achieving multimode imaging-guided hyperpyrexia elimination of tumor.

## Materials and Methods

### Materials

Ethanol, stannum powder (10 μm, 99% trace metals basis), CCK-8 kit, indocyanine green (ICG), 2-(4-amidinophenyl)-6-indolecarbamidine dihydrochloride (DAPI), calcein-AM, and propidium iodide (PI) were purchased from Sigma-Aldrich. Phosphate-buffered saline (PBS, pH 7.4, 10 mM), penicillin–streptomycin, trypsin–EDTA, fetal bovine serum (FBS), and cell culture medium were brought from Gibco Life Technologies (AG, Switzerland). JC-1 Kit was purchased from Cayman Chemical. 1,2-Distearoyl-sn-glycero-3-phosphoethanolamine-N-[methoxy (poly-ethylene glycol)] (DSPE-PEG, MW 5000 Da, PG1-DS-5 k) was obtained from Nanocs, Inc. The other reagents were analytically pure and no need further purification for use. Ultrapure water (18.2 MΩ cm) was applied in all experiments.

### Preparation of SnNSs

2D SnNSs were synthesized by a combination of cryogenic exfoliation and ice-bath liquid-phase exfoliation. In detail, bulk stannum powder (100 mg) was mixed with 40 mL of absolute ethanol in a polyethylene terephthalate (PET) bottle. Afterward, the bottle was immersed in a container containing liquid nitrogen and then the ultrasound probe was inserted into the bottle. The mixed solution was then ultrasonically treated without a stop at 300 W for 5 min. During the sonication, liquid nitrogen was maintained in the container at all times. Then, the bottle containing a mixed solution was transferred to the water bath and ultrasonically treated at 200 W for 5 min. The two processes, cryogenic exfoliation and water bath liquid-phase exfoliation, were repeated nine times in a row. Since the temperature of liquid nitrogen is extremely low, the ethanol in the bottom of the bottle would solidify during the ultrasonic process. Therefore, single liquid nitrogen ultrasound should not be too long. When the surrounding ethanol solution was solidified and the surrounding solution around the probe did not (about 5 min), the mixed solution was transferred to water bath sonication. Afterward, the mixed solution was centrifuged (3000 rpm) for 10 min to collect the SnNSs supernatant. The obtained supernatant was the SnNSs solution and stored in 4 °C for future use.

### Preparation of SnNSs@PEG

To obtain PEGylated SnNSs, SnNSs of ethanol solution (1 mg mL^−1^) were mixed with 20 mL of ethanol solution containing 40 mg DSPE-PEG in equal volume. The obtained mixture was treated by water bath ultrasound for half an hour, and then, the solution was removed via vacuum rotary evaporation. The products were washed twice to remove the excess DSPE-PEG using ultrapure water. The clean SnNSs@PEG were then dispersed into ultrapure water for further use. And the storage temperature of SnNSs@PEG was 4 °C.

### Characterization of SnNSs and SnNSs@PEG

The morphology of SnNSs was characterized by transmission electron microscopy (TEM, JEM-2100F, JEOL, USA). The thickness of SnNSs was detected using atomic force microscopy (AFM; Veeco, NanoMan). The chemical compositions of SnNSs and SnNSs@PEG were examined by Fourier transform infrared spectrophotometer (FTIR) spectra (Nexus 470, Nicolet, Madison, WI, USA) and X-ray photoelectron spectroscopy (XPS, ESCALAB 250, Thermo Fisher, USA). The crystal structure of SnNSs and SnNSs@PEG was determined via X-ray diffraction (XRD, Bruker D8, Germany). Raman spectrum (HORIBA JOBIN YVON, France) was also employed to analyze SnNSs and SnNSs@PEG. A UV–Vis–NIR spectrophotometer (Infinite M200 PRO) was applied to investigate the absorbance of SnNSs@PEG. The concentration of Sn was tested using ICP-AES (7000DV, PerkinElmer).

### First-Principles Calculations of SnNSs

First-principles calculations within the generalized gradient approximation (GGA) were performed by the Vienna ab initio simulation package (VASP) [51, 52]. The generalized gradient approximation (Perdew–Burke–Ernzerhof flavor) was used to treat exchange-correlation potential. The setting cutoff energy of the plane-wave basis was 500 eV and the k-point mesh was k-point mesh in the calculation. The first Brillouin zone was sampled in the Monkhorst–Pack grid. The convergence criterion was given as 1.0 × 10^–6^ eV atom^−1^ for energy and 0.01 eV Å^−1^ for the force during the geometry optimization, respectively. The spin polarization was considered in all calculations.

### Photothermal Effect of SnNSs@PEG

SnNSs@PEG aqueous solutions with different concentrations (0, 25, 50, 100, and 200 μg mL^−1^) were irradiated under an 808-nm NIR laser at different powers (0.5, 0.75 1.0, and 1.25) for 5 min to evaluate their photothermal performance. An IR thermal camera (TI100 Infrared Camera FLK-TI100 9HZ, FLUKE) was used to record the temperature changes during the irradiation.

### Extinction Coefficiency of SnNSs@PEG

Beer–Lambert law was applied to calculate the extinction coefficient of SnNSs@PEG. The equation was described as follows:

1 $$\varepsilon = \frac{{A_{808} }}{{{\text{CL}}}}$$

where *A*_808_ is the absorbance of SnNSs@PEG at 808 nm, *ε* is the extinction coefficient of SnNSs@PEG, *L* is the optical path (cm), and *C* (μg mL^−1^) is the concentration of SnNSs@PEG. According to the above equation, the *ε* was calculated to be 11.23 Lg^−1^ cm^−1^. Compared to some classical PTAs such as graphene oxide (GO, 3.6 L g^−1^ cm^−1^) [53] and AuNRs (3.9 L g^−1^ cm^−1^) [54], the value of this calculation is much higher than them, suggesting the promising potential of SnNSs@PEG for cancer PTT.

### Photothermal Conversion Efficiency (PTCE) of SnNSs@PEG

According to previous reports [45, 55, 56], the calculation steps of the PTCE of SnNSs@PEG are as follows:

Based on the conservation of total energy:

2 $$\sum_{i} m_{i} C_{p,i} \frac{{{\text{dT}}}}{{{\text{dt}}}} = Q_{SnNSs} + Q_{s} - Q_{{{\text{loss}}}}$$

where *m* is the mass of ultrapure water (solvent) and *C*_p_ is the heat capacity of ultrapure water. *T* is the temperature of the solution.

The photothermal energy input of SnNSs@PEG was expressed in terms of *Q*_*SnNSs*_:

3 $$QSnNSs = I(1 - 10^{ - A808} )\eta$$

where *A*_*808*_ is the absorption at 808 nm of SnNSs@PEG, *I* is the power of 808-nm laser, and the PTCE was expressed in terms of *η*.

*Q*_*s*_ is the heat of the solvent, and *Q*_loss_ is the heat lost to the surrounding environment:

4 $$Q_{{{\text{loss}}}} = hA\Delta T$$

where the heat transfer coefficient is expressed in terms of *h*, the surface area of the container is expressed in terms of *A*, and the temperature change of solution is expressed in terms of *ΔT*.

The heat input would be equal to the heat output at the maximum steady state, that is:

5 $$Q_{SnNSs} + Q_{s} = Q_{{{\text{loss}}}} = hA\Delta T\max$$

where the change in temperature at the maximum steady state is *ΔT*_max_. Combining Eqs. 3 and 5, the PTCE (*η*) could be calculated by the following formula:

6 $$\eta = \frac{{hA\Delta T\max - Q_{s} }}{{I(1 - 10^{ - A808} )}}.$$

In order to obtain the *hA*, *θ*, defined as the ratio of *ΔT* to *ΔT*_max_, was therefore introduced:

7 $$\theta = \frac{\Delta T}{{\Delta T\max }}.$$

The following new equation would be got when Eq. 7 was substituted into Eq. 2:

8 $$\frac{{{\text{d}}\theta }}{\theta } = \frac{hA}{{\sum_{i} m_{i} C_{p,i} }}\left[ {\frac{{Q_{SnNSs} + Q_{s} }}{hA\Delta T\max } - \theta } \right].$$

When the laser irradiation was removed, the *Q*_SnNSs_ + *Q*_*s*_ = 0, Eq. 7 would change to the following formula:

9 $${\text{d}}t = - \frac{{\sum_{i} m_{i} C_{p,i} }}{hA}\frac{{{\text{d}}\theta }}{\theta }.$$

Combining Eq. 8 and the followed expression would get:

10 $$t = - \frac{{\sum_{i} m_{i} C_{p,i} }}{hA}\theta.$$

Consequently, using the linear relationship between time and − ln*θ* of the cooling curve, *hA* could be obtained. By substituting the *hA* value into Eq. 6, the PTCE (*η*) of SnNSs@PEG could be calculated.

### Hemocompatibility of SnNSs@PEG

The hemocompatibility of SnNSs@PEG was assessed through hemolysis assay. The specific experimental steps are as follows: First, the erythrocytes were obtained via centrifugation of the mouse blood for 5 min at 4000 rpm. The obtained erythrocyte was then washed with PBS (pH = 7.4) three times for subsequent experiments. Then, red blood cells (100 μL, 8%, v/v) were mixed with 100 μL of SnNSs@PEG solution at various concentrations (50, 100, 200, and 400 μg mL^−1^), PBS, or ultrapure water, respectively. The mixtures were then incubated for 8 h at 37 °C. The supernatants were collected after the centrifugation of the mixed solutions, and then, the absorbance at 540 nm was tested. The hemolysis rate can be calculated according to the following formula:

11 $${\text{Hemolysis}}\,\left( \% \right) = \left( {I/I_{0} } \right) \times 100\%$$

where *I*_0_ represents the absorbance of erythrocytes after complete hemolysis and *I* represents the absorbance of supernatant.

### Cell Culture

4T1, MCF-7, HUVEC, and D511 cells were cultured using DMEM medium at 37 °C in an atmosphere of 5% CO_2_. FBS (10%) and streptomycin/penicillin (1%) were added into all the DMEM medium for adjuvant.

### Cell Uptake Study

The cellular internalization of SnNSs@PEG was investigated by using Cy5.5-labeled SnNSs@PEG. In detail, 4T1 cells were seeded into cell culture dish for 12 h, and then, Cy5.5-labeled SnNSs@PEG were added into cells and continued to incubate for different times (4, 8, and 12 h)*.* Afterward, the medium was removed and the cells were washed with fresh medium twice, and then, the cells were imaged by a laser scanning confocal microscope.

### Cytotoxicity Assessment

CCK-8 assay was applied to investigate the cytotoxicities of SnNSs@PEG to different cell lines. 4T1, MCF-7, HUVECs, and D511 cells were seeded into 96-well plates for 12 h at a density of 1 × 10^4^ cells/well. Then, the medium was removed and SnNSs@PEG were added with various concentrations (25, 50, 100, and 200 μg mL^−1^). After 24- or 48-h incubation, the medium was replaced by the CCK-8 diluent solution. After another culture for 2 h, the absorbance at 450 nm was measured using a microplate reader (Bio-Tek ELx800, USA) to calculate the cell viability.

### In vitro Photothermal Therapy Against Cancer Cells

CCK-8 assay was employed to evaluate the in vitro photothermal therapy of SnNSs@PEG against the tumor. 4T1 cells were selected as model cells to investigate the in vitro photothermal effect of SnNSs@PEG. In detail, 4T1 cells were seeded into 96-well plates for 12 h at a density of 1 × 10^4^ cells/well. Subsequently, the various concentrations of SnNSs@PEG dispersed in culture medium (0, 25, 50, 100, and 200 μg mL^−1^) were added into cells to replace the old culture medium. After another incubation for 4 h, the cells were received the treatment of 808-nm NIR laser (1 W cm^−2^) irradiation for 5 min. After 12-h culture, CCK-8 diluent solution was added into the cells to determine the cell viability. For cells living/dead staining, after the cells received the treatment of irradiation, the cells were cultured for another 0.5 h and then the culture medium containing PI and calcein-AM was added to stain the cells. After 20-min staining, the cells were washed and imaged using an inverted fluorescence microscope.

To further prove the in vitro photothermal effect of SnNSs@PEG against cancer cells, flow cytometry (FCM) was employed to perform cell apoptosis analysis. 4T1 cells were seeded into 48-well plates for 12 h at a density of 2 × 10^4^ cells/well. Afterward, the fresh culture medium containing SnNSs@PEG (200 μg mL^−1^) was added into cells to replace the old medium. After another 4-h incubation, the cells were divided into four groups including G1: control group, G2: NIR laser group, G3: SnNSs@PEG group, and G4: SnNSs@PEG + NIR laser group. For G2 and G4, the cells were treated with NIR laser irradiation for 5 min (1 W cm^−2^). Then, trypsin–EDTA was used to digest the cells after treatments, and the digested cells were collected through centrifugation. The sum of the cells was taken in the five wells as one sample. According to the manufacturer's instructions, the obtained cells were then stained using Annexin V-FITC/PI apoptosis detection kit and analyzed by FCM.

### Mitochondrial Membrane Potential (MMP)

To evaluate the MMP change during the in vitro PTT of SnNSs@PEG against cancer cells, the JC-1 staining assay was performed. In detail, SnNSs@PEG dispersed in the culture medium (200 μg mL^−1^) were co-incubated with 4T1 cells for 4 h. Afterward, the cells were treated with NIR laser irradiation (1 W cm^−2^) for 5 min. As a contrast, the cells that received the treatments of NIR laser alone or SnNSs@PEG only were served as control. Then, the cell culture medium was discarded, and the JC-1 staining solution was added into the cells according to the manufacturer protocol. After 30-min staining, the JC-1 staining solution was removed, and the cells were incubated with a culture medium containing Hoechst 33,342 for another 15 min to stain the cell nuclei. Finally, the cells were imaged by an inverted fluorescence microscope after twice washing.

### Animal Experiments

Healthy BALB/c mice (6 weeks old, male) were brought from Hunan Silaike Experimental Animal Co. Ltd. (Changsha, China). All the animal experiments were executed according to the regulation approved by the Laboratory Animal Center of the Xiangya School of Medicine, Central South University (Changsha, China).

### Establishment of the Xenograft Tumor Model

The mice xenograft tumor model was established by subcutaneously injecting 2 × 10^6^ 4T1 cancer cells on the back of mice. The tumor volume was calculated according to the following formula:

12 $${\text{Tumor volume}}\,\left( {{\text{mm}}^{3} } \right) = 4/3 \times \left( {{\text{tumor width}}/2} \right)^{2} \times \left( {{\text{tumor length}}/2} \right).$$

After the tumor volume was up to ~ 80 mm^3^, the mice could be used in subsequent experiments.

### Pharmacokinetic Studies

Three healthy Balb/c mice were administrated with Cy5.5-labeled SnNSs@PEG (200 μL, 1 mg kg^−1^ equivalent Cy5.5 per mouse) through the tail vein to study the in vivo pharmacokinetic profile of SnNSs@PEG. At different time points, 20 μL of blood was collected and then 180 μL of lysis buffer was mixed with the above blood. Subsequently, a microplate reader was used to measure the fluorescence of mixed solution to examine the concentration of the SnNSs@PEG. In addition, the corresponding standard curve was got through a series of diluents. To eliminate or reduce the blood background auto-fluorescence influence, the blood sample without any treatment was served as a blank sample for the test.

### In vivo Fluorescence Imaging and Biological Distribution Study

To evaluate the in vivo fluorescence imaging of SnNSs@PEG, the mice bearing 4T1 tumor were intravenously injected with 200 μL of Cy5.5-labeled SnNSs@PEG (1 mg kg^−1^ equivalent Cy5.5 per mouse). Then, at different time intervals (1, 2, 4, 8, 12, and 24 h), Maestro2 In-Vivo Imaging of System (Cri Inc.) was used to perform the in vivo imaging. The major tissues including heart, liver, spleen, lung, kidney, and tumor were collected at 12 h post-injection and imaged immediately. Additionally, ImageJ was used to analyze the quantitative fluorescence intensity of major organs and tumors.

### In vivo Photoacoustic Imaging of SnNSs@PEG

To investigate the in vivo photoacoustic (PA) imaging of SnNSs@PEG, the mice bearing 4T1 tumor were received the treatment of administration with 200 μL of SnNSs@PEG (2 mg mL^−1^) through the tail vein. After different times (1, 2, 4, 8, 12, and 24 h) of injection, the mice were euthanized to collect the tumors. The PA imaging was carried out via a PA instrument (LeSonics, Wellman Center of Photomedicine). The PA signals in each region of interest (ROI) were analyzed through the soft of ImageJ.

### In vivo Photothermal Therapy Against Cancer

The mice bearing 4T1 tumor were randomly divided into the following four groups (six mice/group): G1: control group, G2: NIR group, G3: SnNSs@PEG group, G4: SnNSs@PEG + NIR group. Afterward, the mice in G3 and G4 were intravenously injected with 200 μL of SnNSs@PEG (2 mg mL^−1^). At 12-h injection, the mice in G2 and G4 were treated with NIR laser (808 nm, 1.25 W cm^−2^) for 10 min. During the irradiation, the tumor temperature change in the first five minutes was monitored by using an IR thermal camera. Subsequently, tumor volume and mice weight were measured every other day. Additionally, two mice in each group were euthanized to collect the tumors for H&E staining and TUNEL staining on the second day after treatment. According to Eq. (12), the tumor volumes can be obtained. After 14 days of feeding, all the mice were euthanized to harvest the tumors and major organs including heart, liver, spleen, lung, and kidney. The tumors were imaged to confirm the efficacy of photothermal therapy of SnNSs@PEG in vivo. Besides, the collected organs were used for histological analysis. The relative tumor volumes were calculated according to *V/V*_0_, where *V* is the mice tumor volume after 14 days of feeding and *V*_0_ is the original tumor volume of mice.

### In vivo Toxicity Evaluation of SnNSs@PEG

To investigate the in vivo biosafety of SnNSs@PEG, healthy mice were received an intravenous injection of SnNSs@PEG (10 mg kg^−1^). At 1, 7, and 14 d post-injection, the mice were euthanized to collect the blood and major organs including heart, liver, spleen, lung, and kidney. Afterward, the blood biochemistry analysis was executed to confirm the biocompatibility of SnNSs@PEG. The harvested organs were performed for H&E staining to verify the histocompatibility of SnNSs@PEG. In addition, the healthy mice were used as a blank control.

## Results and Discussion

### Characterization of SnNSs and SnNSs@PEG

**Fig. 1:**
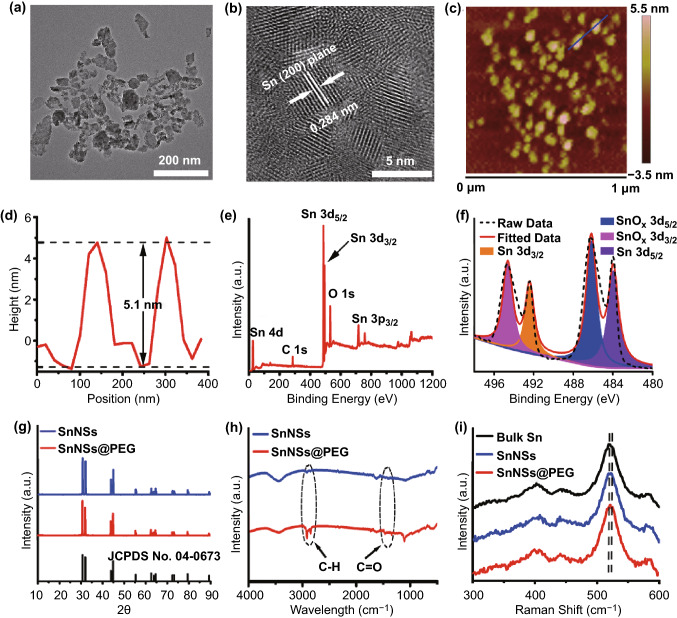
**a** TEM image of SnNSs. **b** HRTEM image of SnNSs. **c** AFM image of SnNSs. **d** Thickness profile of SnNSs in **c**. **e** XPS survey spectrum of SnNSs. **f** XPS spectrum of Sn 3d. **g** XRD profiles of SnNSs and SnNSs@PEG. **h** FTIR spectra of SnNSs and SnNSs@PEG. **i** Raman spectra of Bulk Sn, SnNSs, and SnNSs@PEG

The SnNSs were obtained by the combination of cryogenic exfoliation and liquid-phase exfoliation of Sn powder in ethanol. And a black transparent solution demonstrated the formation of SnNSs (Fig. S1). Transmission electron microscopy (TEM) was employed to characterize the morphology of the synthesized SnNSs. A sheet-like structure of SnNSs was observed, and their average lateral size was about 100 nm (Fig. 1a). The high-resolution TEM (HRTEM) showed that the lattice spacing of obtained SnNSs is about 0.284 nm (Fig. 1b), which is consistent with the (200) plane of Sn [57]. Furthermore, the atomic force microscopy (AFM) image exhibited that the average thickness of SnNSs was about 5.1 nm (Figs. 1c, d and S2), demonstrating that the prepared SnNSs were composed of multiple layers. The chemical composition of SnNSs was determined by X-ray photoelectron spectroscopy (XPS) (Fig. 1e). The 3d_3/2_ and 3d_5/2_ doublets of SnNSs were at 492.36 and 484.01 eV, respectively (Fig. 1f), which are the characteristic peaks of Sn. Additionally, the peaks at 494.69 and 486.18 eV corresponding to the 3d_3/2_ and 3d_5/2_ doublets of SnO_x_ [58] were also observed, indicating the partial oxidation of SnNSs during the preparation. Moreover, since the electronic shielding effect, many bare metal nanoagents are prone to aggregate and precipitate in the salt solution [45, 47]. Consequently, we utilized 1,2-distearoyl-sn-glycero-3-phosphoethanolamine-N-[methoxy (poly-ethylene–glycol)] (DSPE-PEG) to modify the surface of SnNSs for obtaining PEGylated SnNSs (SnNSs@PEG) with high dispersibility and stability in physiological environments. As shown in Fig. S3, after 24-h incubation in different aqueous solutions, negligible agglomeration of SnNSs@PEG was found, whereas obvious aggregation was observed for bare SnNSs in the same conditions, indicating the enhanced stability of SnNSs after PEGylation. Next, Fourier transform infrared (FTIR) spectrum was applied to examine the successful PEGlyation on the surface of SnNSs. The FTIR peaks at 2926 and 1653–1665 cm^−1^ were assigned to the C–H and C=O stretching, respectively, suggesting that the surface of SnNSs was successfully modified by DSPE-PEG. Furthermore, the crystalline structure of SnNSs and SnNSs@PEG was investigated via X-ray diffractometry (XRD). All the XRD peaks of SnNSs and SnNSs@PEG were matched well with the Sn standard card (JCPDS 04-0545), manifesting the high crystallinity of the prepared SnNSs. In addition, no obvious other crystal structures were observed, probably due to the fact that the Sn with oxidation state was an amorphous structure. The Raman spectra also demonstrated the structures of SnNSs and SnNSs@PEG, and a slight redshift was found for the characteristic peak of SnNSs and SnNSs@PEG compared with that of Sn powder. Additionally, similar to other 2D homologous nanomaterials, such as silicene and germanene, SnNSs@PEG displayed broad and strong absorbance from ultraviolet to NIR regions, and a significant concentration dependence was observed (Fig. S4). Overall, these data demonstrated the successful preparation of both SnNSs and SnNSs@PEG.

### Photothermal Performance of SnNSs@PEG

**Fig. 2:**
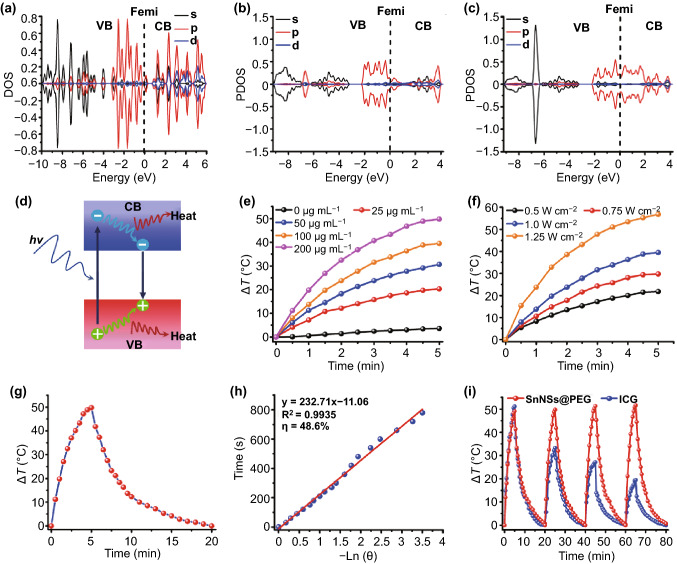
**a** Calculated density of states (DOS) of bulk Sn powder. Calculated projected density of states (PDOS) of SnNSs with three layers: **b** interlayer, **c** bottom and top layer. **d** Schematic illustration of the electron–hole pair generation and relaxation in narrow-bandgap SnNSs under NIR irradiation. **e** Temperature change profiles of SnNSs@PEG solutions at various concentrations (0–200 μg mL^−1^) under NIR laser irradiation (808 nm, 1 W cm^−2^). **f** Temperature change profiles of SnNSs@PEG solution (100 μg mL^−1^) exposed to different power densities (0.5–1.25 W cm^−2^) of NIR laser (808 nm). **g** Temperature increase profile of SnNSs@PEG solution (200 μg mL^−1^) with laser irradiation and cooling profile without irradiation. **h** Linear profile of time versus − lnθ obtained from the cooling period of **g**. **i** Photothermal stability of SnNSs@PEG under NIR laser irradiation with ICG as a control

For photonic nanomedicines, the photothermal performance is crucial to their therapeutic effects against cancer. Thus, the potential photothermal properties of SnNSs were firstly evaluated by first-principles density functional theory (DFT) calculations [59]. Trilayered Sn (200) was built as 2D SnNSs, and bulk Sn was selected as references (Fig. 2a). The middle layer exhibits lower values of density of states (DOS) (Fig. 2b), indicating less available free electrons in the valence channel near Fermi level, which is similar to the electronic characters in bulk Sn near Fermi level. The bulk Sn could therefore be chiefly responsible for light absorption rather than heat transmitting. However, the projected density of states (PDOS) of upper and lower layers shows that the p-orbital of Sn is prominent in the valence band within the same energy range near the Fermi level (Fig. 2c). These free electrons in upper and lower layers may undertake the task of thermal transferring for fewer layered SnNSs, which may cause good photothermal conversion. Additionally, according to the DFT calculations, the theoretical bandgap (*E*_g_) of SnNSs is about 0.387 eV, far less than the energy of photons from near-infrared light (NIR) [59, 60], which may result in the generation of bandgap electron–hole pairs in SnNSs under the NIR irradiation. Subsequently, generated bandgap electrons and holes will relax to the band edges, converting the extra energy into heat via a thermalization process [59] (Fig. 2d).

To confirm the above theoretical calculation results, we next explored the potential photothermal performance of SnNSs@PEG by exposing the aqueous solutions containing various concentrations of SnNSs@PEG to 808-nm laser with different power densities. As shown in Fig. 2e, f, the photothermal performance of SnNSs@PEG exhibited significant concentration and power dependence. When the concentration of SnNSs@PEG was 200 μg mL^−1^ and the power density of NIR laser was 1.0 W cm^−2^, the highest temperature increment (ΔT) of SnNSs@PEG was up to 49.8 °C (Fig. S5). In comparison, the temperature of water only increased by 5.0 °C in the same condition, indicating the excellent photothermal effects of SnNSs@PEG. Moreover, based on the temperature increment curve and the cooling curve of SnNSs@PEG (Fig. 2g), an obvious linear relationship between time and the cooling temperature after transformation was observed (Fig. 2h); thus, the photothermal conversion efficiency (PTCE) of SnNSs@PEG was calculated to be 48.6%. In addition, the NIR extinction coefficient (*β*) of SnNSs@PEG was estimated to be 11.23 L g^−1^ cm^−1^ (Fig. S6), which was calculated based on the Beer–Lambert law (*A*/*L* = *βC*, *A* is the absorption of SnNSs@PEG at the wavelength of 808 nm, *C* is the concentration of SnNSs@PEG, and *L* is the path length) [45, 47, 55]. Notably, both the obtained PTCE (*η*) and NIR extinction coefficient (*β*) of SnNSs@PEG are much higher than other similar nanomaterials such as BP quantum dots (QDs) [61], germanene QDs [47], boron nanosheet [62], antimonene QDs [45], and silicon quantum sheets [63], demonstrating that SnNSs@PEG have great potential to serve as promising photothermal agents. Afterward, the photothermal stability of SnNSs@PEG was evaluated and an FDA-approved photothermal agent, ICG, was used as a comparison. Both SnNSs@PEG and ICG were heated and cooled after four cycles, SnNSs@PEG still exhibited a remarkable photothermal effect, whereas significant temperature reduction was found for ICG (Fig. 2i). The absorption spectra further confirm the above results. As shown in Fig. S7a, the absorbance of SnNSs@PEG did not change obviously after four cycles, suggesting their prominent photostability. By comparison, a remarkable reduction in the absorption of ICG was observed after the same treatment (Fig. S7b), manifesting severe photobleaching of ICG by NIR irradiation. These results make SnNSs@PEG quite promising as photonic nanomedicines.

### Biocompatibility of SnNSs@PEG

**Fig. 3:**
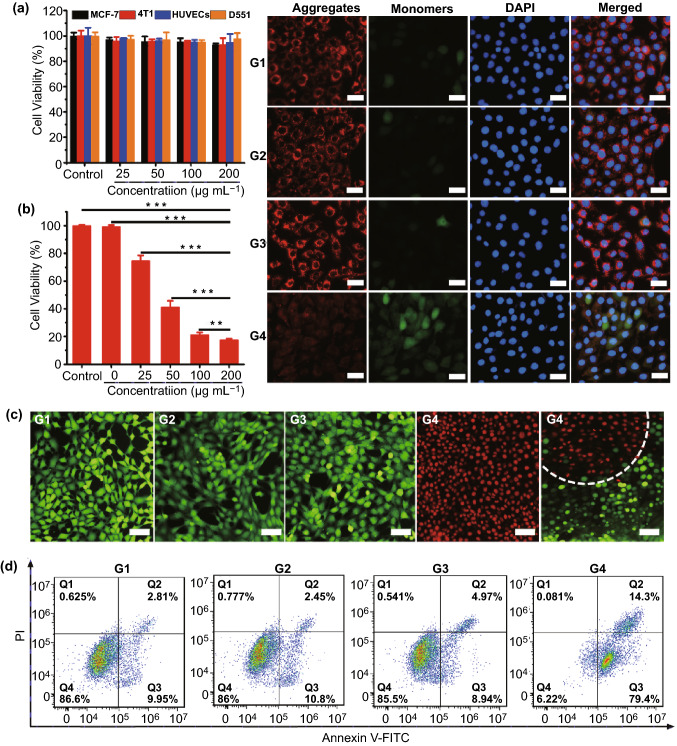
**a** Cytotoxicity of SnNSs@PEG with different concentrations against various cell lines after 24-h incubation. **b** Survival rate of 4T1 cells after treated with SnNSs@PEG and NIR laser (808 nm, 1 W cm^−2^) irradiation for 5 min. **c** Live/dead fluorescence staining images of cells (red: dead, green: live) after different treatments: G1: Control, G2: NIR, G3: SnNSs@PEG, G4: SnNSs@PEG + NIR (scale bar = 25 µm). **d** FCM apoptosis analysis of 4T1 cells after different treatments: G1: Control, G2: NIR, G3: SnNSs@PEG, G4: SnNSs@PEG + NIR. **e** Changes of MMP in 4T1 cells after different treatments: G1: Control, G2: NIR, G3: SnNSs@PEG, G4: SnNSs@PEG + NIR (JC‐1 monomers: green, JC‐1 aggregates: red) (scale bar = 50 µm)

Biocompatibility is another essential consideration for photonic nanomedicines in biomedical applications [64–66]. Therefore, we assessed the hemolysis and cytotoxicity of SnNSs@PEG. As shown in Fig. S8, less than 6.5% hemolysis was observed after red blood cells (RBCs) were co-incubated with SnNSs@PEG for 8 h, indicating the good hemocompatibility of SnNSs@PEG. The cytotoxic results showed that negligible cytotoxicities of SnNSs@PEG toward normal and cancer cells were presented after 24- or 48-h incubation (Figs. 3a and S9). Even when the concentration of SnNSs@PEG was up to 200 μg mL^−1^, the cell survival rate was higher than 87%, indicating the weak cytotoxicity of SnNSs@PEG. These data suggested that SnNSs@PEG have superior biocompatibility for further therapeutic studies.

### In vitro Photothermal Antitumor Activity of SnNSs@PEG

Next, the cellular uptake of SnNSs@PEG was investigated by Cy5.5-labeled SnNSs@PEG. The cellular uptake of Cy5.5-labeled SnNSs@PEG increased over time (Fig. S10), and the cells even showed a distinct red fluorescence after only four hours of co-incubation, reflecting good cellular uptake of SnNSs@PEG by 4T1 cells. Benefiting from the excellent photothermal effect of SnNSs@PEG, we next evaluated their in vitro photothermal antitumor effect against 4T1 cells. After incubation with SnNSs@PEG for 4 h, the 4T1 cells were then irradiated with an 808-nm NIR laser for 5 min. We found that the cell viability gradually decreased with the increase in SnNSs@PEG concentration, and less than 17.5% of cells were alive when the SnNSs@PEG concentration was 200 μg mL^−1^ (Fig. 3b). Afterward, we performed a live/dead cell co-staining experiment to further confirm the prominent photothermal therapeutic effect of SnNSs@PEG against 4T1 cells. As shown in Fig. 3c, a distinct demarcation between dead (red) and live (green) cells was observed, and almost all the 4T1 cells covered by the NIR laser spot lost their activities. In contrast, no obvious cell death was found for the cells treated with NIR laser only or SnNSs@PEG alone (Fig. 3c). Moreover, flow cytometry (FCM) was employed to explore the apoptosis of 4T1 cells mediated by hyperthermia during the photothermal therapy of SnNSs@PEG. In the FCM diagram, we divided the cell population into four areas: living cells (Q4), early apoptotic cells (Q3), late apoptotic cells (Q2), and necrotic cells (Q1). The quantification analysis of the cell population was carried out after the 4T1 cells were received various treatments including PBS (G1), SnNSs@PEG alone (G2), NIR laser only (G3), and SnNSs@PEG + NIR (G4). As shown in Fig. 3d, extensive cell apoptosis was presented after the cells were treated with SnNSs@PEG + NIR, and the majority of apoptotic cells were in early and late apoptosis, while negligible apoptotic cells were found for the other control groups.

Subsequently, to explore the photothermal antitumor mechanism of SnNSs@PEG, cell mitochondrial membrane potential (MMP) changes during the photothermal therapy were examined through a JC-1 staining kit [47]. Previous reports confirmed that the cell apoptosis was closely related to mitochondrial dysfunction [67], and JC-1 would form aggregates with red fluorescence for the normal cells mitochondrial due to their high MMP. On the contrary, owing to the decreased MMP, the mitochondrial in damaged cells would be stained by green fluorescent JC-1 monomer [67]. As expected, the 4T1 cells showed bright red fluorescence, which is similar to the control cells, after they received the treatments of SnNSs@PEG alone or NIR laser only, and the ratio of green fluorescence to red fluorescence (G/R) was only about 0.11 (Fig. 3e). However, for the 4T1 cells that suffered from SnNSs@PEG and NIR laser treatment, both the green fluorescence and red fluorescence were observed, and their ratio of G/R increased to 1.8, indicating the dysfunctional mitochondria with reduced MMP in the 4T1 cells. Hence, we speculated that the local hyperthermia generated from the photothermal effect of SnNSs@PEG caused mitochondrial dysfunction, thereby resulting in the change of MMP and cell apoptosis.

### In vivo Multimode Imaging of SnNSs@PEG

**Fig. 4:**
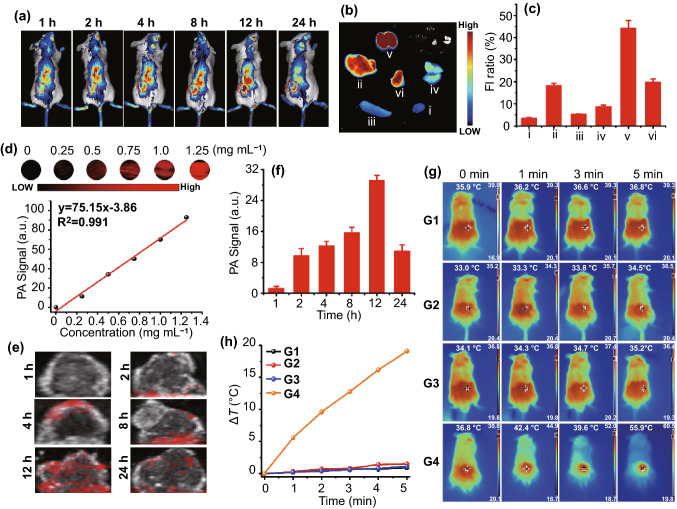
**a** In vivo fluorescence images of tumor-bearing mice at different time points after tail vein injection of Cy5.5-labeled SnNSs@PEG. **b** Ex vivo fluorescence image of tumor and major organs at 12 h after the injection of Cy5.5-labeled SnNSs@PEG. (i) heart, (ii) liver, (iii) spleen, (iv) lung, (v) kidney, (vi) tumor. **c** Relative fluorescence intensity quantification of different organs and tissues in **b**. **d **In vitro PA imaging of SnNSs@PEG solutions at different concentrations and linear fitting curve between SnNSs@PEG concentration and PA signal intensity. **e** PA imaging of the mice tumors at different time points after injection of SnNSs@PEG. **f **Quantitative analysis of PA signal intensity in **e**. **g **Photothermal imaging of mice tumors during different treatments (G1: Control, G2: NIR, G3: SnNSs@PEG, G4: SnNSs@PEG + NIR). **h** Temperature change curve of mice tumor sites during various treatments

Encouraged by these positive in vitro results, we next investigated the in vivo behavior of SnNSs@PEG. The in vivo biodistribution of Cy5.5-labled SnNSs@PEG in 4T1 tumor-bearing mice was first explored. As shown in Fig. 4a, after intravenous administration of 4T1 tumor-bearing mice with SnNSs@PEG, the accumulation of Cy5.5-labled SnNSs@PEG in tumor regions gradually increased over time, and the highest accumulation was observed after 12-h injection. The ex vivo image further revealed the fluorescence distribution in tumor and primary organs (Fig. 4b). The imaging result can be seen from Fig. 4c although most Cy5.5-labeled SnNSs@PEG were accumulated in the kidney, about 19.9% of the fluorescence could still be found in the tumor, which was probably attributed to the enhanced permeability and retention (EPR) effect. The pharmacokinetic results also confirmed the relatively long blood circulation time of SnNSs@PEG, which was consistent with the biodistribution results described above (Fig. S11). In general, high NIR absorption could produce strong photoacoustic (PA) signal [47], and we thus applied PA imaging to further assess the tumor accumulation of SnNSs@PEG. Notably, significant concentration dependence for the PA signal of SnNSs@PEG was found (Fig. 4d). Moreover, there was an obvious linear relationship between the PA signal and concentration. After the tumor-bearing mice were intravenously administrated with SnNSs@PEG, the PA imaging was then executed at different time intervals. Similar to the NIR fluorescence imaging results, the PA signals also gradually increased with the increase in time and reached their peak at 12 h post-injection; the signal then faded (Fig. 4e, f), demonstrating the high accumulation effect of SnNSs@PEG by the EPR effect.

### In vivo Photothermal Antitumor Activity of SnNSs@PEG

**Fig. 5:**
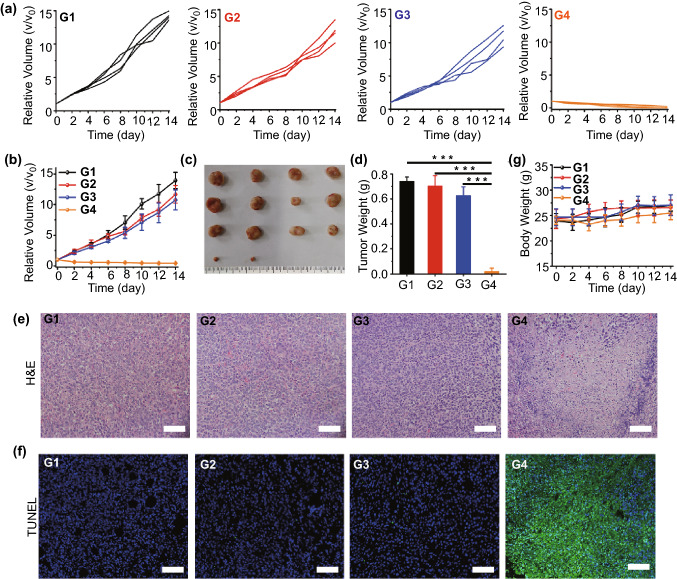
**a** Individual tumor growth profiles: (G1) control, (G2) NIR, (G3) SnNSs@PEG, (G4) SnNSs@PEG + NIR. **b** Average tumor growth profiles for all treatment groups. **c** Digital images of tumors in after various treatments. **d** Average tumor weight for all treatment groups. **e** HE and **f** TUNEL staining of tumor slices after the treatments (scale bar = 40 μm). **g** Body weight changes in each group during treatment

Finally, the in vivo therapeutic effect of SnNSs@PEG was evaluated. The tumor-bearing mice were randomly divided into four groups including control groups (G1), NIR groups (G2), SnNSs@PEG groups (G3), and SnNSs@PEG + NIR (G4). For G3 and G4, the mice were intravenously administrated with SnNSs@PEG, and the mice of G1 and G2 were intravenously injected with saline. Then, the mice of G2 and G4 were irradiated with an 808-nm NIR laser at 12 h post-injection. Additionally, the temperature of tumor sites was recorded using an IR thermal camera to evaluate the in vivo photothermal effect of SnNSs@PEG during the irradiation. As displayed in Fig. 4g, the temperature of the tumor only increased by 0.9 °C after 5-min irradiation for control group mice. In sharp contrast, for the mice of G4, the temperature increment of tumor sites was over 19.1 °C (Fig. 4h), manifesting superior photothermal performance of SnNSs@PEG in vivo. Furthermore, rapid tumor growth was presented for G2 and G3, which was almost the same as that of the control group (Fig. 5a), suggesting that SnNSs@PEG alone or NIR laser only could not suppress tumor growth. Conversely, for the mice of G4, their tumors were nearly completely ablated after PTT, and no obvious recurrence was emerged (Fig. 5b, c). Histological analysis including H&E **(**Fig. 5d) and TUNEL (Fig. 5e**)** staining further verified that the vast majority of the cells in the tumor tissue were damaged and apoptotic for the mice of G4, while the tumor cells of the other groups still maintained their normal morphology and no obvious changes were found, suggesting the prominent in vivo photothermal treatment effect of SnNSs@PEG. Notably, there was no significant change in mice body weight compared to the control groups (Fig. 5g), and no obvious tissue damage for the major organs (heart, liver, spleen, lung, kidney) was detected during the treatment (Fig. S12).

To investigate the biocompatibility of SnNSs@PEG in vivo, a toxicological evaluation was performed systematically. After the healthy mice were administrated with SnNSs@PEG through intravenous injection, euthanasia of mice was executed at 1, 7, and 14 days post-injection to harvest the major organs and blood for histological section staining and blood biochemical analysis, respectively. We found that their blood indicators were almost the same as control mice, which were in the normal ranges (Fig. S13). Furthermore, during the treatments, the major organs also exhibited negligible histological toxicity and side effects (Fig. S14), indicating the excellent in vivo biosafety of SnNSs@PEG. Taken together, these exciting results demonstrated that the photonic nanomedicine of SnNSs@PEG with superior biocompatibility, remarkable photothermal performance, good multi-mode imaging capability, and excellent PTT efficacy holds great potential for cancer theranostics.

## Conclusions

In summary, 2D SnNSs were synthesized through an approach combining cryogenic exfoliation and liquid-phase exfoliation, which were further developed as photonic nanomedicines for cancer theranostics. The obtained SnNSs@PEG with a high PTCE of 48.6% exhibited excellent photothermal effects, good stability, and superior biocompatibility. Importantly, guided by the multimode imaging (NIR fluorescence/PA/photothermal imaging) of SnNSs@PEG, precise and effective tumor ablation could be achieved by photothermal therapy. Given largely unexplored of 2D stannum in biomedical applications, this study greatly expands the application prospects of 2D SnNSs though rationally designing their multifunctionality and exploring the related physicochemical properties, especially on cancer phototherapy. Meanwhile, this research may open up a promising avenue to further explore the 2D stannum as nanomedicine for different bioapplications. For instance, owing to the unique 2D layered structure of SnNSs, we believe they could serve as drug carriers with high drug loading (similar to black phosphorus, germanene, or antimonene) for more effective treatment of various diseases including cancer. As a new paradigm, we believe these highly effective photonic nanomedicines based on 2D SnNSs with facile synthesis and superior biocompatibility are promising in biomedical applications.

## Supplementary Information

Below is the link to the electronic supplementary material.

Supplementary file1 (PDF 1158 KB)
