# Supplementary material for: Cryogenic Exfoliation of 2D Stanene Nanosheets for Cancer Theranostics
Source: Nanomicro Lett. 2021 Mar 10;13:90. doi: 10.1007/s40820-021-00619-1 (PMC8006518; doi:10.1007/s40820-021-00619-1)
Supplement: Supplementary file 1 — Supplementary file1 (PDF 1158 KB) [file 40820_2021_619_MOESM1_ESM.pdf]

Supporting Information for

Cryogenic Exfoliation of 2D Stanene Nanosheets for Cancer Theranostics

Jiang Ouyang[1, 2, 3, #], Ling Zhang[3, #], Leijiao Li[4], Wei Chen[1], Zhongmin Tang[1], Xiaoyuan Ji[1], Chan Feng[1], Na Tao[3], Na Kong[1], Tianfeng Chen[2, *], You-Nian Liu[3, *], Wei Tao[1, *]

[1]Center for Nanomedicine and Department of Anesthesiology, Brigham and Women's Hospital, Harvard Medical School, Boston, MA 02115, USA

[2]The First Affiliated Hospital, Department of Chemistry, Jinan University, Guangzhou 510632, P. R. China

[3]College of Chemistry and Chemical Engineering, Central South University, Changsha, Hunan, 410083, P. R. China

[4]School of Chemistry and Environmental Engineering, Changchun University of Science and Technology, Changchun 130022, Jilin Province, P. R. China

[#]J. Ouyang and L. Zhang contributed equally to this work

*Corresponding authors. E-mail: tchentf@jnu.edu.cn (T. F. Chen); liuyounian@csu.edu.cn (Y.-N. Liu); wtao@bwh.harvard.edu (W. Tao)

Supplementary Figures and Table

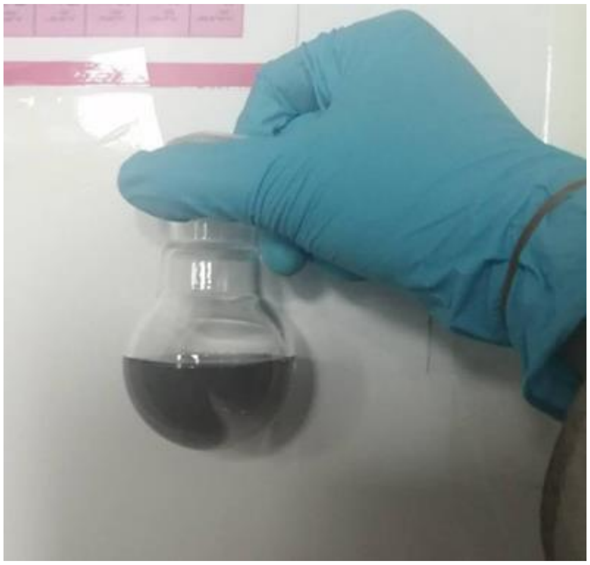

Fig. S1 Digital images of obtained SnNSs after exfoliation

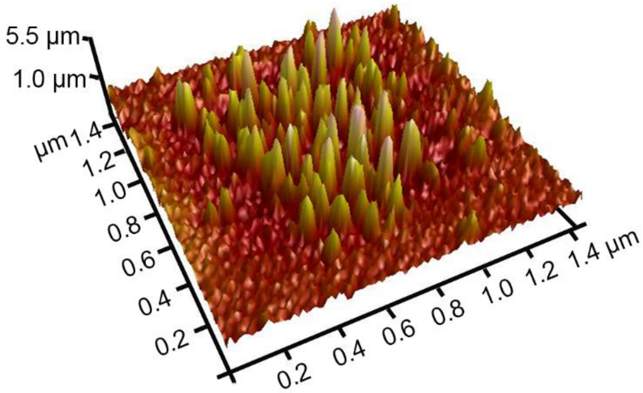

Fig. S2 3D AFM image of SnNSs

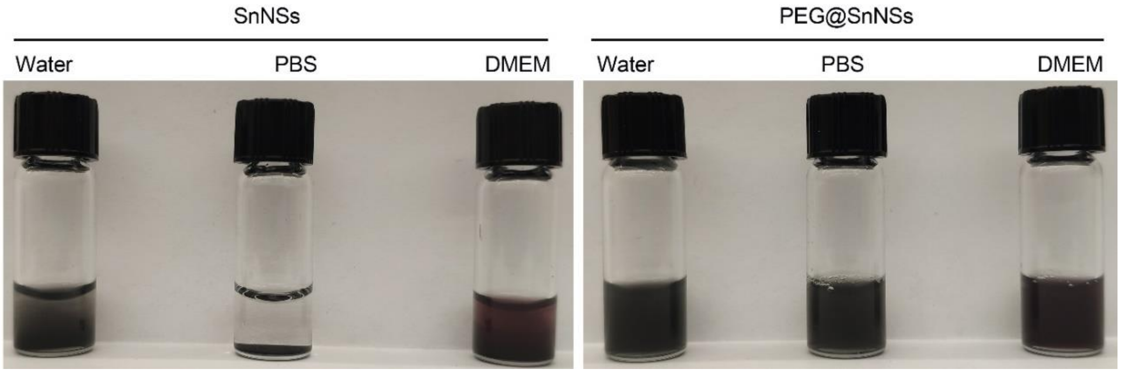

Fig. S3 Dispersity of SnNSs and SnNSs@PEG in water, PBS, and DMEM medium after 24 h incubation

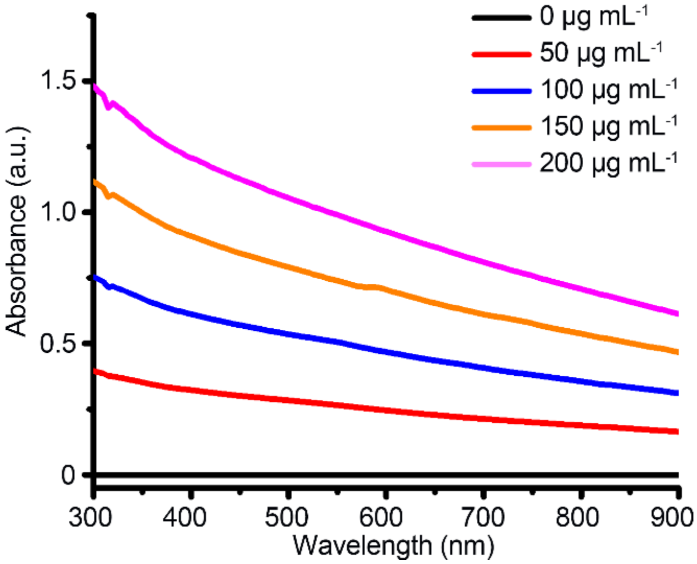

Fig. S4 Absorbance spectrum of SnNSs@PEG solution with various concentrations

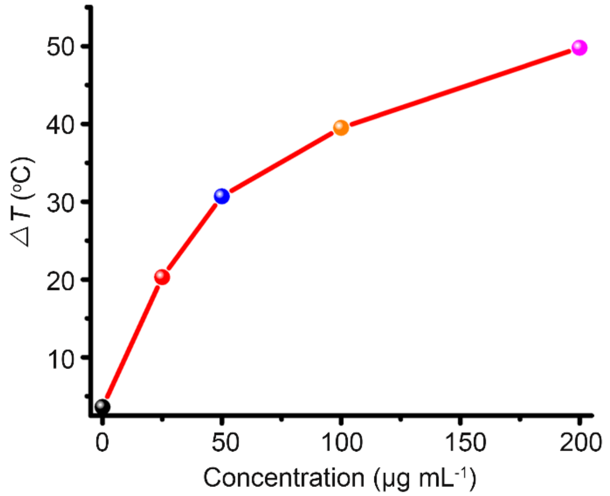

Fig. S5 Temperature increment (ΔT) of SnNSs@PEG solutions with various concentrations after 5 min laser irradiation

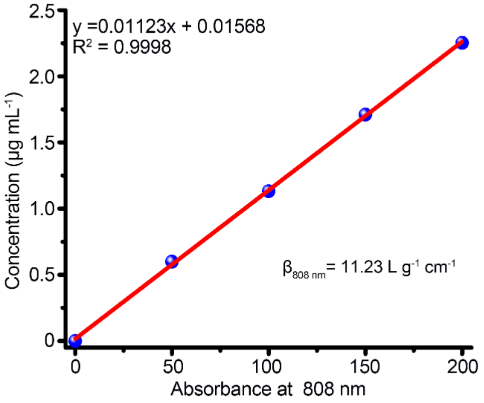

Fig. S6 Normalized absorbance intensity of SnNSs@PEG divided by the optical distance (A/L) at various concentrations for λ=808 nm

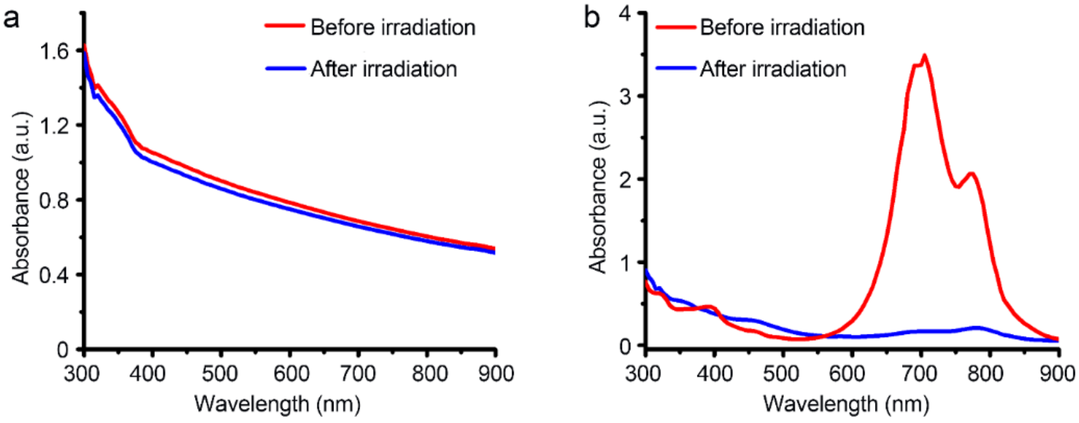

Fig. S7 Absorbance spectrum of (**a**) SnNSs@PEG and (**b**) ICG before and after NIR laser irradiation

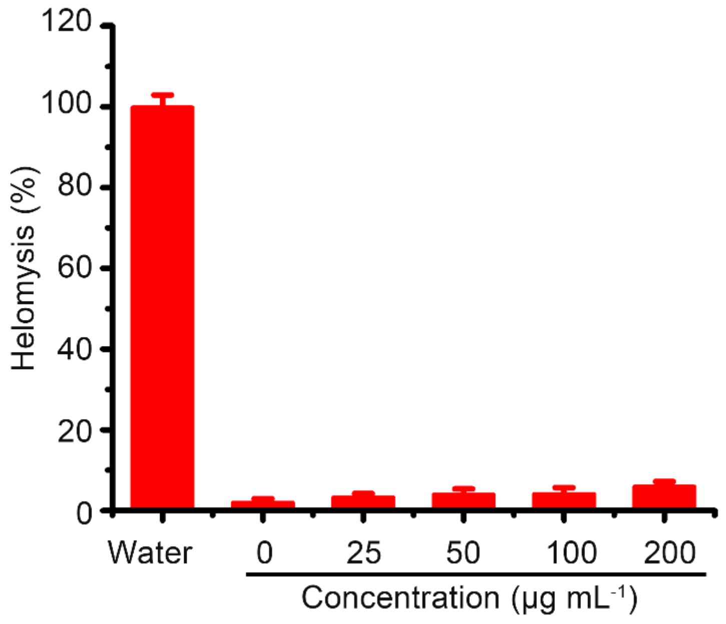

Fig. S8 Hemolysis of red blood cells (RBC) at various concentrations of SnNSs@PEG

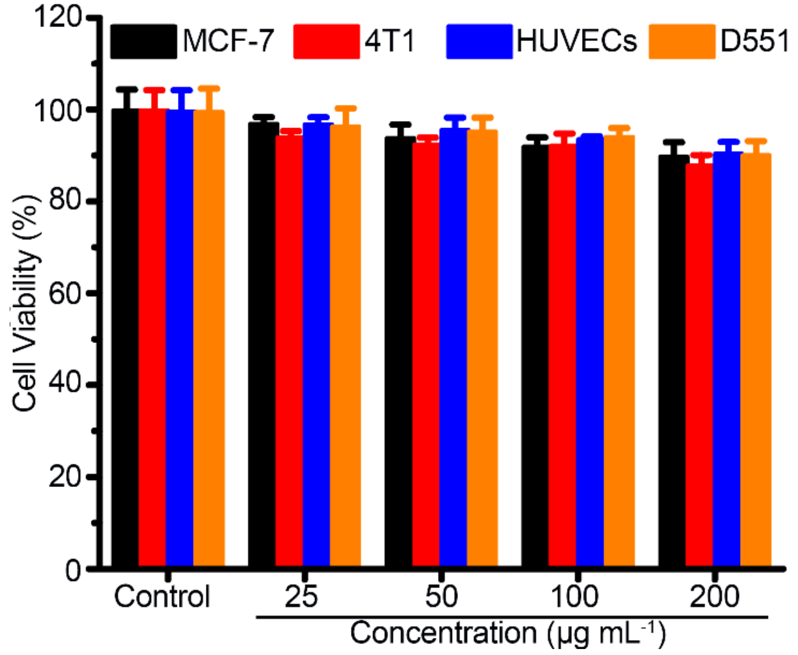

Fig S9 Cytotoxicity of SnNSs@PEG against various cell lines at different concentrations after 48 h incubation

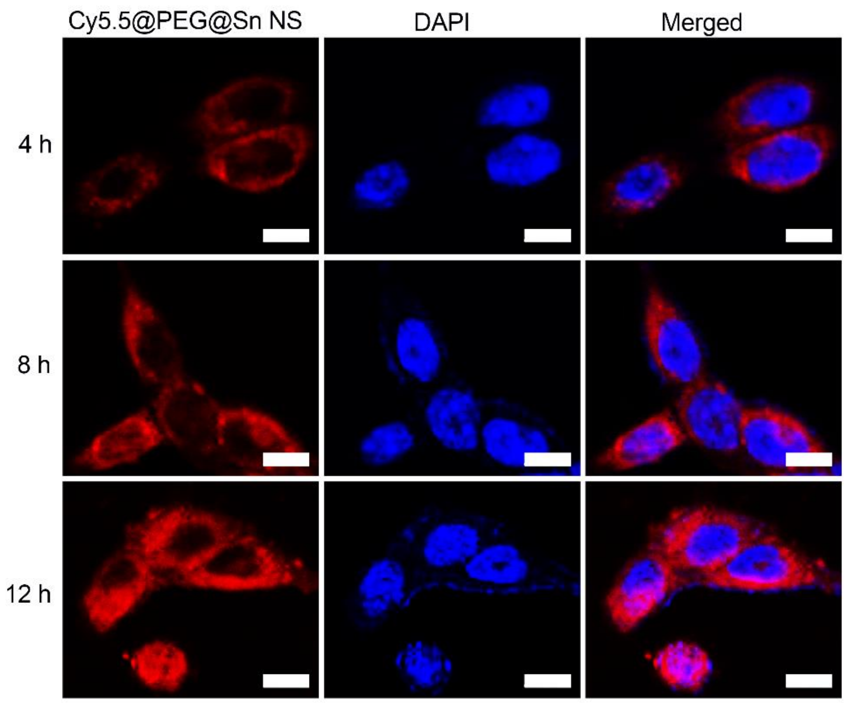

Fig. S10 Cell uptake of SnNSs@PEG (Scale bar = 40 μm)

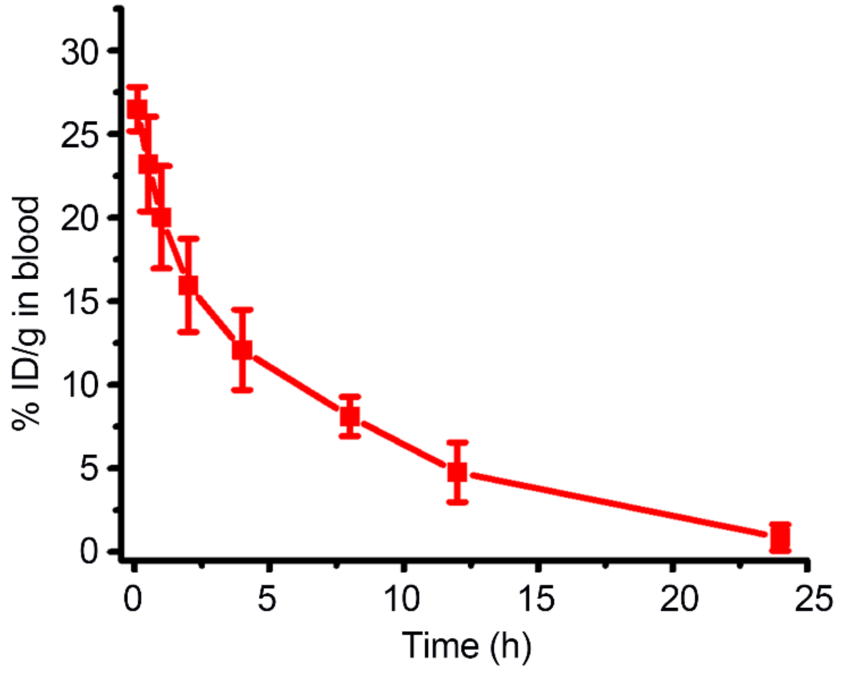

Fig. S11 Blood circulation profile of Cy5.5-SnNSs@PEG determined by absorption spectrum

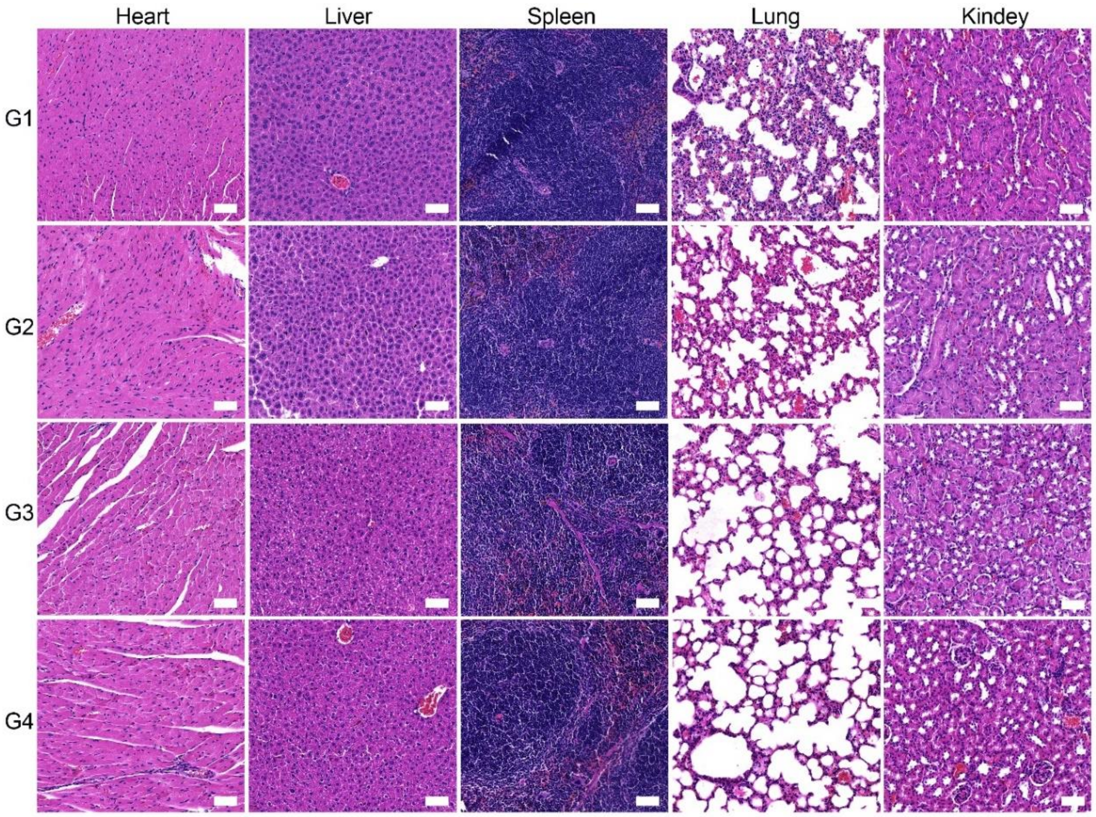

Fig. S12 H&E staining of main organs after variuos treatments: G1: control, G2: NIR, G3: SnNSs@PEG, G4: SnNSs@PEG + NIR (Scale bar = 50 μm)

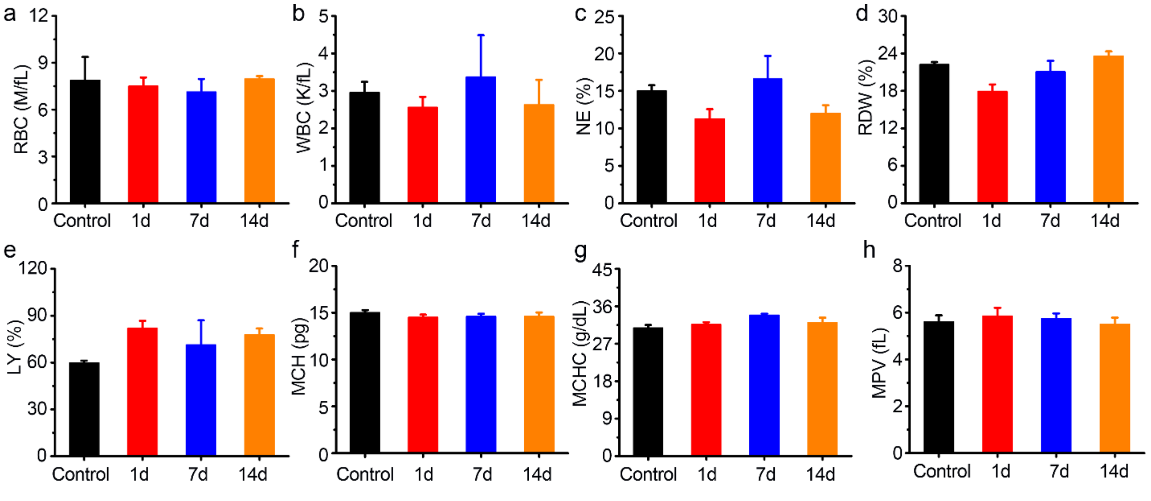

Fig. S13 Blood biochemistry analysis of the mice after intravenous injection of SnNSs@PEG (20 mg kg^{-1}) at different intervals: (**a**) Red blood cells, (**b**) White blood cells (WBC), (**c**) Neutrophilic granulocyte percentage (NE%) (**d**) Red blood cell distribution width (RDW), (**e**) Lymphocyte Percentage (LY%), (**f**) Mean corpuscular hemoglobin (MCH), (**g**) Mean corpuscular hemoglobin concentration (MCHC), (**e**) Mean platelet volume (MPV)

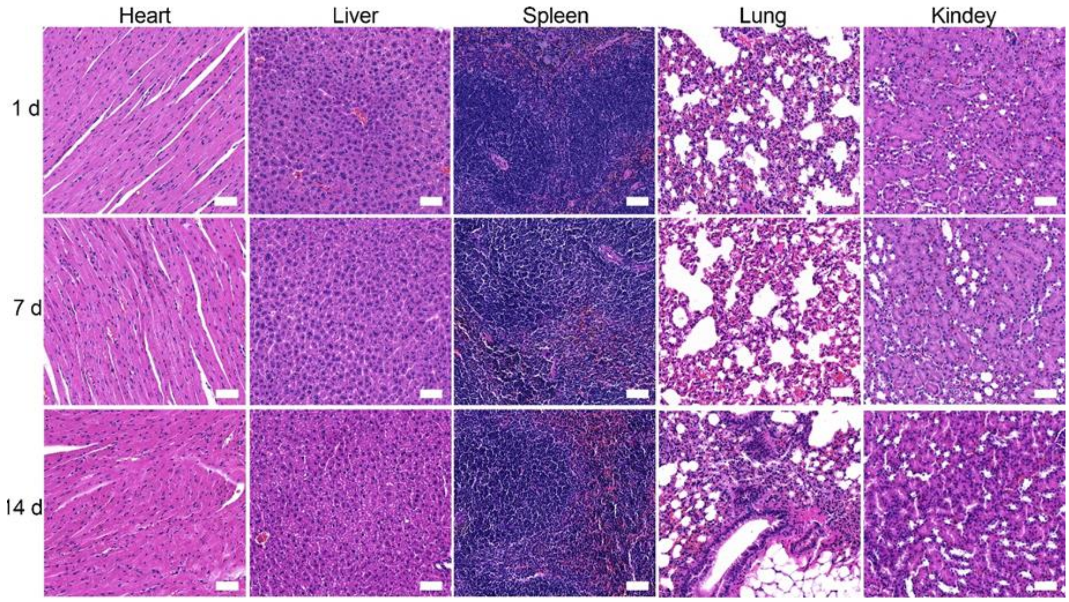

Fig. S14 H&E staining of main organs from mice at different days post injection with SnNSs@PEG (Scale bar = 50 μm)

Table S1 Photothermal conversion efficacy of several photothermal agents at 808 nm laser irradiation

Photothermal agents	Photothermal conversion efficacy (%)	References
Au nanorods	22.1	Angew. Chem. Int. Ed. 2013, 125, 4263-4267
Polypyrrole nanoparticles	45	Chem. Commun., 2012,48, 8934–8936
Dopamine-melanin colloidal nanospheres	40	Adv. Mater. 2013, 25, 1353-1359
WS_2 nanosheets	32.8	Nanoscale,2014, 6,10394–10403
MoS_2 nanosheets	24	ACS Nano 2014, 8, 6922-6933
BP quantum dots	28	Angew. Chem. Int. Ed. 2015, 54, 11526-11530
Bi_2Se_3 nanosheets	34.6	Small 2016, 12, 4136–4145
WSe_2 nanosheets	35.1	J. Mater. Chem. B, 2017, 5, 269--278
Antimony quantum dots	45.5	Angew. Chem. Int. Ed. 2017, 56, 11896-11900
Ti_3C_2 nanosheets	30.6	Nano Lett. 2017, 17, 384−391
Boron nanosheet	42.5	Adv. Mater. 2018, 30, 1803031
Germanene quantum dots	45.9	Angew. Chem. Int. Ed. 2019, 138, 13539-13544
Silicon quantum sheets	47.2	Sci. Bull. 2021, 66, 147-157
